# Motivation, Cues to Action, and Barriers to COVID-19 Vaccine Uptake: A Qualitative Application of the Health Belief Model among Women in Rural Zambia

**DOI:** 10.4269/ajtmh.24-0005

**Published:** 2024-08-27

**Authors:** Kayla J. Kuhfeldt, Jeanette L. Kaiser, Allison J. Morgan, Thandiwe Ngoma, Davidson H. Hamer, Günther Fink, Peter C. Rockers, Benson Chirwa, Nancy A. Scott

**Affiliations:** ^1^ Department of Global Health, Boston University School of Public Health, Boston, Massachusetts;; ^2^ Right to Care Zambia, Lusaka, Zambia;; ^3^ Section of Infectious Diseases, Boston University Chobanian & Avedisian School of Medicine, Boston, Massachusetts;; ^4^ National Emerging Infectious Diseases Laboratory, Boston University, Boston, Massachusetts;; ^5^ Center on Emerging Infectious Diseases, Boston University, Boston, Massachusetts;; ^6^ Swiss Tropical and Public Health Institute and University of Basel, Basel, Switzerland

## Abstract

Vaccine hesitancy has played a major role in slowing the global COVID-19 response. Using cross-sectional, primarily qualitative data collected in four rural districts in Zambia, we aimed to explore community perceptions of COVID-19 disease and vaccines, including perceived motivators, cues to action, benefits, and barriers to vaccine uptake as guided by the Health Belief Model. In-depth interviews (IDIs) were conducted in late 2021 with women of reproductive age who were enrolled in an early childhood development study. Although two-thirds of the 106 respondents reported low perceived risk of catching COVID-19, they expressed concern that the COVID-19 pandemic had impacted their daily lives and feared effects of the disease. They had generally positive beliefs that the vaccine would be accepted among their communities when it became more widely available. Reported motivators to vaccine uptake included desire for protection against COVID-19 and understanding vaccine purpose, due to ongoing education from health personnel, neighbors, friends, radio, and church leaders. Misinformation or reported bad experiences served as cues away from vaccine uptake. Examples of misinformation included the vaccine causing COVID-19 or another disease and death and vaccines being associated with the devil and against Christian beliefs. Accounts of pain after receiving the vaccine also discouraged uptake. Perceived benefits included a desire to be protected from the disease, belief in the effectiveness of the vaccine, fear of catching COVID-19, and belief the vaccine would limit negative effects. Health system implementers and policy makers should consider recipient motivators and cues to action to further increase vaccination rates.

## INTRODUCTION

The COVID-19 pandemic was declared a Public Health Emergency of International Concern by the WHO in January 2020 and has created lasting impacts globally.^1^^,^^2^ Vaccination, as a strategy to address the ongoing COVID-19 pandemic, has played a vital role in reducing the severity of COVID-19 disease. However, vaccine hesitancy has played a major role in slowing global COVID-19 vaccine efforts. Lack of knowledge, mistrust, and a lack of equitable resources have contributed to the growing vaccine hesitancy issue globally.^3^ The resulting low vaccine coverage has left populations in low- and middle-income countries vulnerable to COVID-19.

COVID-19 was first identified in the sub-Saharan African country of Zambia on March 18, 2020.^4^ Similar to countries worldwide, the Zambian National Public Health Institute established guidelines to minimize the spread of COVID-19 throughout the country, which included mandated testing in specific locations as well as widespread use of non-pharmaceutical interventions, such as the use of face masks, limiting gathering sizes, handwashing, and quarantining when exposed to the virus or symptomatic.^4^ Zambia experienced multiple COVID-19 infection peaks in August 2020, January 2021, and November 2021.

In April 2021, the Ministry of Health (MoH) in Zambia developed a national deployment and vaccination plan for COVID-19 vaccines in line with the November 2020 WHO & UNICEF Interim Guidance, which sought to bring comprehensive COVID-19 vaccine knowledge to the eligible population and reduce vaccine refusal rates to less than 5% by the end of 2021.^5^ Vaccination was voluntary in Zambia. The first doses were provided to at-risk individuals, including healthcare workers, traditional leaders, clergy, personnel at borders, police, and security personnel.^6^ Zambia used multiple platforms to encourage citizens to be vaccinated to increase uptake and widespread protection within the country.^7^ However, Zambia’s large-scale vaccination campaign that brought the vaccine to rural areas did not commence until December 2021.^8^ Low uptake of the vaccine was an initial concern among stakeholders; Zambia’s neighbor, the Democratic Republic of the Congo, returned more than 1 million doses to the vaccine producer due to low uptake.^9^^,^^10^

Zambia’s MoH has had a history of successful vaccination campaigns, including a measles-rubella vaccine campaign in 2020.^11^ However, the global acceptance of the COVID-19 vaccine is unclear as vaccine hesitancy remains a major barrier for vaccine campaigns globally^9^^,^^11^^–^^14^ and the acceptance of the COVID-19 vaccine in Zambia, particularly in harder to reach rural areas, is not fully known.^12^^,^^14^

Within the endline data collection (October–November 2021) for a cluster randomized controlled trial testing the impact of an early childhood development intervention in two rural Zambian provinces, we included qualitative questions to women of reproductive age on COVID-19 risk behaviors and vaccines. Guided by the Health Belief Model, we assessed vaccine hesitancy among this population by analyzing the community’s and individual’s perceptions of COVID-19 protection behaviors and the vaccine, along with reported barriers and facilitators to vaccine uptake.

## MATERIALS AND METHODS

### Study setting.

Zambia, a lower-middle income country in southern Africa, has a population of 19.6 million, the majority (60%) of which resides in rural areas.^15^ The COVID-19 pandemic’s negative impacts on employment, income, and infrastructure, likely compounded the existing poverty, gender discrimination and inequality within the country.^16^^,^^17^ Generally, Zambia has a young population with nearly 50% of the population under age 15 and only 3% of the population over age 65.^18^ Zambia also has a high burden of infectious diseases including HIV/AIDS (11% prevalence among adults 15–59) and tuberculosis (455 cases per 100,000 population).^18^^,^^19^ However, the health system in rural Zambia is limited by human and financial resource constraints.^5^

The overarching evaluation was conducted in the primarily rural districts of Choma, Kalomo, and Pemba districts of Southern Province and Nyimba district of Eastern Province. Each province has a population of approximately 2 million, with population densities ranging from 28 to 36 people per square kilometer.^15^ These populations generally have little access to electricity, improved water sources, or improved sanitation; the majority are smallholder famers.^18^ Educational attainment is low, particularly among women, although higher in the study provinces than in other Zambian provinces.^18^

### Theoretical framework.

**Figure 1.**
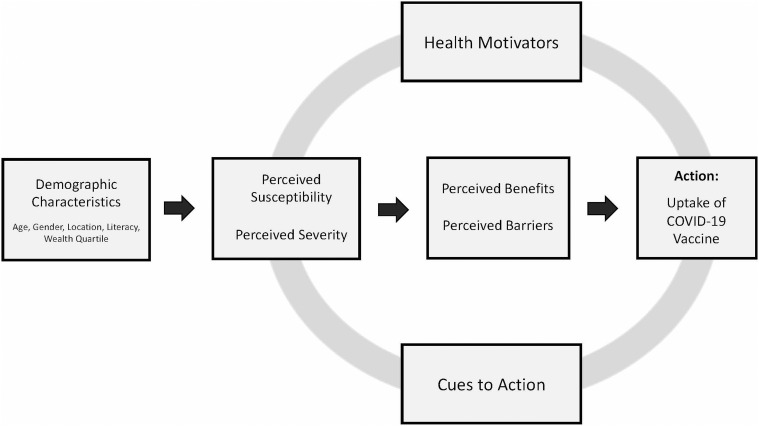
The Health Belief Model. Adapted from Rosenstock (1977).^20^

We interpret the findings of this study through the Health Belief Model, a conceptual model developed in the early 1950s by social scientists at the U.S. Public Health Service that illustrates how individuals use and understand preventative health behaviors and their failure to adopt disease prevention strategies.^20^ The Health Belief Model consists of four main constructs: perceived susceptibility, perceived severity, perceived benefits, and perceived barriers, all of which influence an individual’s likelihood of taking a specific action to prevent illness (Figure 1). The Health Belief Model has been used previously to understand influenza vaccination uptake and is becoming one of the most widely used models for COVID-19 vaccinations.^13^^,^^21^^–^^24^ The Health Belief Model constructs have been recognized as important predictors for the action of receiving a vaccine.^13^^,^^25^^,^^26^ Although the Health Belief Model does not factor in availability of vaccines as an opportunity, this is a known limitation, which we adapted and accounted for to fit this research scenario.

For this study, the Health Belief Model was adapted to fit the data collected. The Health Belief Model constructs typically indicate that if an individual has a high perceived susceptibility to (or risk from) catching a disease and perceives the severity of the disease to be high (i.e., medical or social consequences), the individual would be more likely to take a certain preventative health action. Women in our study were asked about their perceived risk or level of concern regarding COVID-19 and how COVID-19 has disrupted their daily lives.

For this adaption of the Health Belief Model, health motivators were considered the intrinsic factors that led an individual to a certain decision. We explored community perceptions of health motivation through the reported facilitators that make receiving a COVID-19 vaccine easier for someone in the community. Cues to action are the events, people, or external factors that trigger a person to change their behavior. We explored cues to action through their individual reasons for getting the vaccine.

We also explored perceived benefits of receiving a COVID-19 vaccine and the barriers that make it difficult to receive a COVID-19 vaccine through the reported individual thoughts on getting the vaccine themselves and the community perceptions of perceived barriers (Figure 1).

### Study design.

This is an analysis of cross-sectional, qualitative-dominant mixed methods data captured as part of a cluster-randomized trial assessing the effectiveness of community-based parenting groups on child development conducted in the catchment areas of 10 rural health centers. At the endline observation of the trial, we administered household surveys to caregivers enrolled in the parent trial and in-depth interviews (IDI) to a subset of those caregivers. We added a module on COVID-19 vaccine perceptions to the IDI guide. Information on the intervention and overarching study design is publicly available from clinicaltrials.gov (ID no. NCT03991182).

### Sampling.

In August–September 2019, primary female caregivers to 1,108 infants under 6 months of age were systematically sampled for the baseline household survey. First, villages within each health zone were selected with probability proportional to population size. All eligible households were identified by community-based health volunteers, randomly ordered by the study team, and visited systematically. Three or more eligible households were selected from within each village, with an expected average of 15 households per health zone. The same cohort was followed 2 years later in October–November 2021, with successful follow-up made at 84% of households (*n* = 934). An endline household survey was conducted with all sampled women; approximately 10% were also randomly selected for a semi-structured IDI.

### Instruments and measures.

The IDI guides contained COVID-19-related questions, including respondent level of concern regarding COVID-19, self-protection behaviors, and impact of COVID-19 on their daily life (i.e., schooling, group attendance, visiting a health center, social support, and stress). Additionally, one set of questions focused on COVID-19 vaccine information, concerns, community perceptions of the vaccine, and barriers and facilitators to getting the vaccine. Probes were used to elicit more information. The IDI guides were translated into the local languages of Chitonga and Chinyanja for use in Southern and Eastern Provinces, respectively. Supplemental Material 1 includes the portion of the IDI guide related to COVID-19.

Woman and household-level characteristics were collected though the household survey at baseline and confirmed at endline. Woman-level characteristics included respondent literacy, which was assessed by asking the respondent to read as much as they could of one of four sentences in the language of their choice using the 2018 Zambian Demographic and Health Survey (DHS) literacy card.^18^ Household-level characteristics included a short series of asset questions from the Zambian DHS.^18^

In the endline survey, we asked study respondents about their self-perceived risk of catching COVID-19. This was measured quantitatively on a 5-point Likert scale from very low to very high.

### Data collection methods.

Study staff trained a team of short-term local data collectors, who were fluent in the relevant local languages. The data collectors were trained on research ethics, principles of qualitative data collection, and on the data collection instruments. Data collection occurred over a 6-week period in October and November 2021. Transmission of COVID-19 was lower at this point, and prevention measures (face masks, social distancing, and use of alcohol-based hand sanitizers) were followed to limit the possibility of transmission during data collection activities. All IDIs were audio-recorded with the consent of the respondent.

## STATISTICAL ANALYSES

Audio-recordings from the IDIs were translated into English and concurrently transcribed in Microsoft Word^®^ by individuals fluent in English and one or both local languages. A mixed inductive-deductive approach was used for coding the transcriptions. A codebook was initially created based on the questions in the instruments including levels of concern, information on COVID-19, self-protection, exposure, impact on daily life, and information on the vaccine. Participants quantitatively indicated their perceived risk and were aggregated into perceived risk categories of low, moderate, and high during analysis. Content analysis was conducted using matrix queries to compare responses for each code by perceived risk category (low/moderate/high) to identify themes. Due to similarity in responses for all themes, qualitative responses have been presented in aggregate below rather than by perceived risk category. We summarized dominant as well as divergent themes and included illustrative quotes in tables. All coding and analysis were conducted in NVivo Release 1.7.1 (QSR International, Doncaster, Australia).

Demographic data were cleaned and analyzed in SAS v9.4 (SAS Institute Inc., Cary, NC). Descriptive characteristics for location, age, marital status, education, literacy, wealth quintile (constructed based on household assets), and perceived risk from COVID-19 are presented as means and standard deviations (SD) or percentages.

## RESULTS

### Perceived susceptibility and severity to COVID-19.

In total, 106 women participated in the IDI in 2021. Respondents were 30 years old on average, married or cohabiting, and had completed an average of 7 years of formal education (Table 1). The majority of respondents were fully literate. Approximately 80% of the respondents reported low or moderate self-perceived risk of catching COVID-19, whereas 23% felt they were at high risk.

**Table 1:** Demographic characteristics of the IDI respondents

Demographic Characteristics	Total IDI Sample (*N* = 106)
District, *n* (%)	
Choma/Pemba	39 (36.8)
Kalomo	51 (48.1)
Nyimba	16 (15.1)
Age (in years), mean (SD)	30.1 (8.0)
Married/cohabiting, *n* (%)	86 (81.1)
Highest grade completed, mean (SD)	7.0 (3.1)
Literacy, *n* (%)*	
Cannot read at all	31 (29.2)
Literate (can ready part of sentence)	9 (8.5)
Literate (able to read whole sentence)	66 (62.3)
Wealth quintile, *n* (%)	
Lowest—1	22 (20.8)
2	25 (23.6)
3	21 (19.8)
4	15 (14.2)
Highest—5	22 (20.8)
Perceived COVID-19 risk, *n* (%)^†^	
Low	70 (66.0)
Moderate	12 (11.3)
High	24 (22.6)

IDI = in-depth interviews; SD = standard deviation.

* Literacy was assessed at endline by asking respondents to read as much as they could of one of four sentences in the language of their choice using the 2018 Zambian Demographic and Health Survey literacy card.

^†^ The following question was asked, with responses given on a 5-point Likert Scale: “In your opinion, what is your risk of catching COVID-19 disease?”

The IDI respondents generally described being concerned, scared, or worried about COVID-19 (Table 2). Respondents described people not adhering to prevention measures as a cause for their concern. They reported high perceived severity of the disease as causing concern, COVID-19 could result in death, and the disease is easy to catch because it is transmitted through the air.

**Table 2:** Themes and illustrative quotes for perceived susceptibility and severity of COVID-19 expressed by in-depth interviews respondents

Themes	Illustrative Quotes
Concerned, scared, or worried about COVID-19: - Disease is deadly/fear death - Easy to catch COVID-19 because it is transmitted through the air - People not following guidelines/unable to follow guidelines	“I am concerned because they say this disease kills. So, I am concerned because I don’t want to die.” —*Female, age 41, Kalomo district, very low perceived risk* “It concerns me because this virus kills, and it has no cure.” *—Female, age 24, Kalomo district, very low perceived risk* “My concern is that some [people] do not wear masks. Maybe when you meet or in families there is no hand sanitizer or soap or ash or maybe they do not have water. Others do not even sit one meter apart. That is my biggest concern because I may end up getting [COVID-19].” *—Female, age 23, Kalomo district, high perceived risk*
Not concerned about COVID-19: - Religious beliefs are protection enough - Have not experienced the disease firsthand - Low population density means COVID-19 will not spread in rural areas	“No, I don’t feel [at risk]. … [I am not concerned] because God is bigger than everything. … God is the one who takes care of us.” *—Female, age 22, Choma district, very low perceived risk* “I am not concerned … because I have not yet seen a person suffering from COVID-19. We just hear that people are sick.” *—Female, age 36, Kalomo district, very low perceived risk* “We cannot get COVID here because it is hot and we live far from each other … we are not concerned about corona.” *—Female, age 21, Nyimba district, moderate perceived risk*

Only a few respondents reported not feeling concerned about COVID-19, explaining that their faith would protect them, not seeing COVID-19’s impact, or feeling less at risk because of the low population densities of rural areas or that they knew how to protect or take care of themselves.

The majority of respondents noted that the COVID-19 pandemic had changed their daily lives, including the need to perform prevention behaviors (washing hands, wearing face masks in public, inability to gather with family/friends), which influenced whether and how people attend gatherings, such as church, clinics, and schools. Respondents discussed children not being able to attend school, effects on their businesses, and the increasing daily anxiety/worry they felt. A few illustrative quotes are as follows:

*The children stopped going to school. They faced a challenge because I do not know how to read. Secondly, this child … didn’t finish injections [for routine child immunizations]. They refused us from going to the clinic. Thirdly, they stopped us from going to church and gatherings of our group meetings. That was the challenge. Then I also get very worried. —Female respondent, age 37, Choma district, very low perceived risk.*

*Coronavirus has affected businesses, [and] no movements [are allowed]. People just observe social distance by sitting 1 meter apart. So, we are affected. There is no peace. —Female, age 29, Nyimba district, very high perceived risk.*

### Health motivation for COVID-19 vaccine uptake.

Two main motivators were described by respondents for uptake of the COVID-19 vaccine: 1) need for protection against COVID-19 echoing perceived risk and disease severity and 2) understanding about vaccine purpose due to ongoing education (Table 3). Additionally, respondents theorized that widespread availability to the vaccines at community centers, such as health posts and schools, would allow for greater access to the vaccine and influence community motivation. When these motivators are in place, respondents described community members being more likely to get the COVID-19 vaccine. Conversely, some respondents felt their communities would reject the vaccines because of their misconceptions about it.

**Table 3:** Themes and illustrative quotes for health motivation to COVID-19 vaccines as expressed by in-depth interviews respondents

Themes	Illustrative Quotes
Desire for protection: - Desire to be protected from the disease - Fear effects of catching COVID-19	“Yes, many people want to be vaccinated … because they want to be protected from the disease. They have all witnessed how it disturbs and kills people.” *—Female, age 40, Kalomo district, low perceived risk* “They can want to be vaccinated because if one suffered from COVID-19 its very far from where the COVID isolation centers is for one to access treatment.” *—Female, age 22, Choma district, moderate perceived risk* “This pandemic is deadly, so people want to protect themselves.” *—Female, age 23, Kalomo district, high perceived risk* “Because they are scared of getting sick.” *—Female, age 29, Nyimba district, very high perceived risk*
Knowledge: - Understanding vaccine purpose is important for uptake	“What can make it easier is teaching them because there are people who just hear about this vaccine and covid and still thing it is not really there. Teaching them to say let us all work together and get vaccinated to get protected from this disease so that id finishes meaning everyone can be encouraged knowing what they need to do.” —*Female, age 29, Kalomo district, high perceived risk.* “Going to the hospital, they first teach you, when they finish teaching you they give you guidelines, about how the COVID-19 disease has come. Then, when you also follow the instructions, you feel that let me also get vaccinated so that I am protect I get a good life I manage to grow my family.” *—Female, age 31, Kalomo district, very low perceived risk*
Availability: - Widespread availability would allow for easy access to the vaccine	“If the vaccine is brought near people in the nearest health posts, it can be easier to get this protection from there.” *—Female, age 19, Choma district, high perceived risk.* “What can make it easier to get vaccinated against COVID-19 is the availability of the vaccine. Once they announce at school that the vaccine is there and you gather to get vaccinated, it can be easier that way.” *—Female, age 35, Kalomo district, very low perceived risk*

### Cues to action for COVID-19 vaccine: sources of information.

Respondents reported multiple sources of vaccine information that motivated their decision toward or away from getting the vaccine (Table 4). A few information sources supporting vaccination included health personnel, neighbors, friends, the radio, and church leaders. However, community members, friends, or health personnel who provided misinformation (or bad experiences) about the vaccine served as cues away from the vaccine. Examples of misinformation included that the vaccine would cause death by causing the brain to mix with the blood. Accounts of pain after receiving the vaccine also discouraged some from getting it.

**Table 4:** Themes and illustrative quotes for sources of Cues to Action to COVID-19 vaccines as expressed by in-depth interviews respondents

Themes	Illustrative Quotes
Cue towards vaccine: - Health Personnel - Neighbors - Friends - Radio - Church leaders	“The health personnel encouraged us to get vaccinated. They said we should get vaccinated because if you are not vaccinated you will not be allowed to mingle with other people.” *—Female, age 35, Kalomo district, very low perceived risk* “It was my neighbor … he said if you are vaccinated, even if another wave comes and you get it, it will not be powerful on you because you will be protected.” *—Female, age 54, Kalomo district, very low perceived risk.* “After listening on radio, I went to the clinic to consult. After consulting that when I got vaccinated.” *—Female, age 32, Nyimba district, very low perceived risk* “The leaders of Siachitema mission, they are the ones who encouraged me because I did not know anything. When I went to get treated, they told me that I need to get the COVID-19 vaccine which protects you from COVID. That is when I made a decision to get vaccinated to protect myself from COVID-19.” *—Female, age 29, Kalomo district, high perceived risk*
Cue away from vaccine: - Friends - Health personnel - Community members	“It’s just friends … The thing they said, they said when you get this injection, for you to be protected, the brain will come out, the brain will mix with the blood. … We feared that even before your time comes to die, you will die. That’s what scared us.” *—Female, age 40, Kalomo district, very high perceived risk* “What would make it hard is the discouraging information given by some health workers. Others who got vaccinated would be telling people that the injection is very painful, or others say, ‘When I got injected, I got swollen’ or ‘When I got vaccinated, I got sick’ so that discourages people.” *—Female, age 23, Kalomo district, high perceived risk*

### Perceived benefits of receiving COVID-19 vaccines.

Perceived benefits of the vaccines included wanting to be protected from the disease (believing in the effectiveness of the vaccine) and fear over the effects of catching the disease (believing the vaccine would limit these negative effects; Table 5). Some respondents also indicated that the vaccine would allow them to resume daily activities that COVID-19 had impacted, such as leaving the home.

**Table 5:** Themes and illustrative quotes for perceived benefits from COVID-19 vaccines as expressed by in-depth interview respondents

Themes	Illustrative Quotes
Vaccines are effective: - Want to be protected from disease - Fear effects of catching COVID-19	“We desired to have good health. That’s why we chose to be vaccinated against COVID-19.” *—Female, age 43, Kalomo district, moderate perceived risk* “It’s easy because we want to be protected against the disease. That’s why it’s easy for us, it’s the desire to be protected.” *—Female, age 23, Kalomo district, very low perceived risk* “I was encouraged to get the COVID-19 vaccine because I cannot know if I can be in contact with someone or contract the disease so let me get protected so that even when the disease comes it will be weak in me. Why I choose that is so that I find protection in my life with COVID-19.” *—Female, age 29, Kalomo district, high perceived risk*
Reduced impact on daily life: - Allows to resume daily activities	“What made us to choose to get vaccinated against COVID-19 is that we heard those whose are not vaccinated are not supposed to move about. They should be restricted to their homes. Now what kind of a life is that one.” *—Female, age 35, Kalomo district, very low perceived risk* “Because I know that I am already protecting myself when I am moving, like wearing masks, and social distancing. I believed all these things first things.” *—Female, age 34, Kalomo district, very low perceived risk*

### Perceived barriers to COVID-19 vaccine acceptability and uptake.

Three major barriers emerged: 1) lack of knowledge of the vaccines and misconceptions, 2) fear or worry about the vaccines, and (3) unavailability of vaccines (Table 6). Respondents described their communities not knowing the purpose of the vaccine, holding misconceptions that there was no proof of the effectiveness of the vaccines in preventing COVID-19, and that the vaccines are not only ineffective but causes someone to come down with COVID-19. Misinformation on the post-vaccine symptoms and effects (including death) and/or belief that the COVID-19 vaccines are associated with the devil or are against Christian beliefs drove fear of the vaccines. Additionally, the continuously evolving context proved difficult. Furthermore, a few respondents reported community members doubted the reality of COVID-19, believing it to be a lie.

**Table 6:** Themes and illustrative quotes for perceived barriers to COVID-19 vaccine acceptability and uptake as expressed by in-depth interview respondents

Themes	Illustrative Quotes
Lack of knowledge: - Don’t understand purpose of vaccines - No proof of vaccine effectiveness - Doubt reality of COVID-19 - Believe COVID-19 is a lie	“The vaccine they inject you [with], we do not have proof that it is for COVID-19 so everyone should stand on their own.” *—Female, age 37, Choma district, low perceived risk* “Some say, ‘We cannot get vaccinated because COVID-19 pandemic is over, so there is no reason to get vaccinated,’ ‘The vaccine is ensuring that COVID-19 continues’ just like that.” *—Female, age 41, Kalomo district, very high perceived risk* “Others do not believe that this disease is real. … They just think that it is for lies.” *—Female, age 26, Choma district, very high perceived risk*
Misinformation drives fear of vaccines: - Vaccines are associated with the devil or are against Christian beliefs - Vaccines cause COVID-19 or another disease - Vaccines cause death	“The majority of people just think that once they get the injection, they will be infected by Coronavirus.” *—Female, age 42, Kalomo district, moderate perceived risk* “Mostly we think that when you get vaccinated, you are getting another disease. So, there is a lot of fear.” *—Female, age 37, Choma district, moderate perceived risk.* “No [the community] cannot accept [the vaccine] … because we are told the moment that you get vaccinated, that is when you get the virus, and you will die.” *—Female, age 24, Kalomo district, very low perceived risk* “Some are willing, but others are saying [the vaccine] is satanic. They say if you put something where you have been injected it will be stuck. That is what people are telling us. For example, they say, ‘You put a phone where you have been injected it will get stuck also, they say you have received 666.’” *—Female, age 44, Nyimba district, moderate perceived risk*
Unavailability of vaccines: - Few places provide vaccines - Far distance to hospitals to get vaccine	“What can just be difficult because sometimes you go to get vaccinated then you find that is has finished. So, that has brought hardship.” *—Female, age 24, Kalomo district, high perceived risk* “If they don’t bring it [to our villages] for us, the distance to walk to the hospitals is far.” *—Female, age 31, Pemba district, low perceived risk*

Some respondents indicated that it was hard to access the vaccines because of low supplies in health centers and the distance they must travel to reach the centers. However, others noted that there were no barriers to accessing the vaccines in their communities.

### Recommendations for increasing COVID-19 vaccine acceptability and uptake.

Respondents provided two main recommendations for increasing COVID-19 vaccine acceptability and uptake: 1) improve education about the COVID-19 vaccines and 2) increase availability of the vaccines. The majority of respondents noted that if communities were better educated about the COVID-19 vaccine and encouraged with the correct information, more in the community would be willing to accept it. This would facilitate uptake of the vaccine generally. Additionally, the majority of respondents noted that there was a need for increased access to the COVID-19 vaccines. Having the vaccine readily available to all communities would reduce barriers to getting vaccinated and increase uptake.

## DISCUSSION

In this article, we explore perceptions of COVID-19 disease and vaccinations among a population of rural Zambian female caregivers. We aimed to interpret results through the Health Belief Model, which considers perceived susceptibility, perceived severity, perceived benefits, and perceived barriers, all of which influence an individual’s likelihood of taking a specific action to prevent illness, within the overarching context of motivators and cues to action. To date, only a few studies have explored the perceptions from the perspectives of community members in Zambia, and none did so at the household level.

During the COVID-19 pandemic, governments faced the unprecedented task of rapidly managing widescale supply—procuring, distributing, and administering doses for their full populations—while concurrently attempting to manage factors that affect demand, including the quality and reach of information and the implementation of sensitization efforts to minimize hesitancy and maximize uptake. Contextually, data for this article were collected when Omicron was beginning to be the predominant circulating variant globally but had not yet hit its peak in Zambia and was prior to Zambia’s large-scale vaccination campaign that brought the vaccine to rural areas in December 2021.^8^ At the time, vaccines were also more widely available in low- and middle-income countries than before. Also, the Zambian national deployment and vaccination plan for COVID-19 vaccines was in line with the November 2020 WHO & UNICEF Interim Guidance, which sought to bring comprehensive COVID-19 vaccine knowledge to the eligible population and reduce vaccine refusal rates to less than 5% by end of 2021.^5^ This increase in knowledge and community experience in receiving the vaccine could be a powerful means of dispelling rumors and misinformation on the vaccines’ effectiveness and side effects.

In terms of susceptibility and severity, we surprisingly found that quantitatively, 66% of our respondents reported low or very low perceived risk of catching COVID-19, but qualitatively reported feeling scared, worried, and concerned about how it would affect their lives. A sensitivity analysis of the qualitative data showed no substantive or meaningful differences in qualitative results when stratified by quantitative risk category. Although the rural areas are generally the most challenging to reach with information, our previous work showed that the Zambian government was able to reach remote areas through television, radio, and social media campaigns and by engaging community leaders to support sensitization.^27^ Vaccine hesitancy has consistently been found throughout sub-Saharan Africa and the Global South more generally.^28^ This has continued to be true for the COVID-19 vaccine. In late 2021, female Zambian respondents in this study indicated persisting concern over the COVID-19 pandemic, including still fearing the effects of the disease and still feeling at risk due to its transmissibility.

When considering the COVID-19 vaccine, intrinsic motivation included the perceived benefits, such as wanting to be protected from the disease, believing in the effectiveness of the vaccine, fear over effects of catching the COVID-19, and believing the vaccine would limit these negative effects. Similar to other literature,^29^ motivations and cues to action overall were often influenced by intrinsic factors such as personal or interpersonal experiences. The majority of respondents believed their communities accepted or would accept the vaccines and explained strong motivators toward vaccination, including widespread knowledge due to ongoing education from health personnel, neighbors, friends, radio, and church leaders. A recent scoping review found that vaccine hesitancy was persistent among healthcare workers in Democratic Republic of Congo, Ghana, Nigeria, and Ethiopia.^30^ Although not specific to Zambia, this is potentially concerning given that much of the information delivered in the remote areas of Zambia comes from health workers. However, several qualitative studies conducted in Zambia suggest that health workers have generally accurate information and perceptions of the virus,^27^^,^^29^ which ideally will translate to also having accurate information and perceptions regarding the vaccine.^31^ A scoping review also found that fear of adverse events as well as misconceptions were barriers to vaccine uptake across many African countries.^30^ Corroborating this, our results suggest that when participants heard or knew of misinformation or reported bad experiences, it served as a cue away from vaccine uptake. Misinformation included notions that the vaccine would cause the brain and blood to mix, resulting in death; that the vaccine causes COVID-19 or another disease; and that the vaccines are associated with the devil and are against Christian beliefs. Accounts of pain after receiving the vaccine also discouraged uptake.

External, particularly structural factors also influenced decision-making and perceptions of vaccines. Most significantly, our results suggest that the potential of widespread access to vaccines at community distribution centers would be motivating, but at the time of the study, vaccines were not widely available at the community level. This is consistent with other studies that report inconvenient clinic hours, wait times, and location; cost of treatment; and insufficient vaccine supply as barriers to vaccination.^32^^,^^33^

Overall, this qualitative study suggests that our sample of remote-living, rural women were generally well informed about COVID-19, and they believed that there would be generally high uptake of the vaccine when or if it were to become more widely available. The government vaccination campaign commenced in December 2021, 2 months after these data were collected. As of November 2023, data suggests that 64% of the population in Zambia had received at least one dose of the vaccine^34^; data specifically from rural areas are not available.

### Limitations.

This study had several limitations. First, these were purely qualitative data, and responses were subject to desirability bias, although this was likely mitigated by generally asking about community perceptions rather than individual actions. Second, these data were collected just before the Omicron variant became widespread, and although vaccines were becoming widely available in Zambia, they were not yet widely accessible to our population. As such, we did not capture vaccination status of our respondents, which would have been extremely low. Further research on vaccine rates in these rural communities and their current vaccine perceptions is warranted. Third, some respondents in this study participated in the study’s parenting groups, which met twice monthly and, after April 2020, included messaging around COVID-19 transmission prevention and adherence to government guidelines. We analyzed data by study arm but found no differences in perceptions. Lastly, respondents in this study had a mean age of 30 years because they were primarily the mothers of children around 2 years of age. These data are unable to provide the perspectives of individuals most at risk of adverse complications from COVID-19, including the elderly or immunocompromised, critical targets for the COVID-19 vaccine campaigns. Studies from the Global South specifically targeting their vaccine uptake rates and perceptions would be particularly valuable.

## CONCLUSION

This study adds to a growing body of literature around vaccine perceptions among community-level participants in rural Africa, contributing the perspectives of remote-living women of reproductive age. Factors that emerged from these data regarding motivators and cues to action should be considered to increase vaccination rates further.

## Supplemental Materials

10.4269/ajtmh.24-0005 Supplemental Materials
