# Supplementary material for: Motivation, Cues to Action, and Barriers to COVID-19 Vaccine Uptake: A Qualitative Application of the Health Belief Model among Women in Rural Zambia
Source: Am J Trop Med Hyg. 2024 Aug 27;111(5):1118–26. doi: 10.4269/ajtmh.24-0005 (PMC11542508; doi:10.4269/ajtmh.24-0005)
Supplement: Supplemental Materials [file tpmd240005.SD1.pdf]

Supplementary File 1: In-depth Interview Guide Questions
The below questions were included in the IDI guide under the COVID-19 theme; responses to these questions were included in the analysis for this article.

1. Tell me, how concerned are you about getting COVID-19?

Follow-up questions:

i) Why do you feel that way?
ii) How likely do you think it is?
iii) How severe do you think it would be?

2. What do you hear about COVID-19 from people in your community that you don't believe or that you think is wrong?

3. How do you protect yourself from catching COVID-19?

4. Did you, someone in your family, or someone close to you ever had or were exposed to COVID-19? If so, please tell me the story about what happened and what the outcome was.

5. How, if at all, has COVID-19 impacted your daily life? For example, think about school, health visits, stress, attendance at social gatherings/church/parenting groups, etc.

Follow-up questions (Ask for things they don't address initially):

i. How it affected your children going to school? Please give an example.
ii. How has it affected your attendance to groups/meetings? Please give an example.
iii. How has it affected your likelihood of visiting the health center? Please give an example.
iv. How has it affected your well-child visits to the clinic? Please give an example.
v. How has it affected your social support? Please give an example
vi. How has it affected your stress? Please give an example

6. What have you heard about the COVID-19 vaccine(s)?

Follow-up questions:

i. Have you heard anything that worries you?
ii. Who did you hear this from?
iii. Do you think it's true? Why?

7. Have you thought about getting a COVID-19 vaccine? What did you decide? Please explain why you made that decision.

Follow-up Questions:

i. Who influenced your decision about whether or not to get the COVID-19 vaccine? (Family and friends, religious and community leaders, HF staff)
ii. What did they say that influenced you?
iii. What other factors influenced your decision about whether or not to get the COVID-19 vaccine? (Availability of vaccine, distance, perception of risk, close experience)

8. Would most people in your community want to get the vaccine if it were easily available? Why or why not?

9. What would make it easier for you or people in your community to get a COVID-19 vaccine?

10. What would make it harder for you or people in your community to get a COVID-19 vaccine?
